# Advances in Carcinogenic Metal Toxicity and Potential Molecular Markers

**DOI:** 10.3390/ijms12129576

**Published:** 2011-12-20

**Authors:** Preeyaporn Koedrith, Young Rok Seo

**Affiliations:** 1 Department of Life Science, Dongguk University, 30 Pildong-ro 1-gil (26 Pildong 3-ga), Jung-gu, Seoul 100-715, Korea; E-Mail: pkoedrith@gmail.com; 2 Institute of Environmental Medicine for Green Chemistry, Dongguk University, 30 Pildong-ro 1-gil (26 Pildong 3-ga), Jung-gu, Seoul 100-715, Korea

**Keywords:** carcinogenicity, DNA damage, DNA repair, genotoxicity, heavy metal, oxidative stress

## Abstract

Metal compounds such as arsenic, cadmium, chromium, cobalt, lead, mercury, and nickel are classified as carcinogens affecting human health through occupational and environmental exposure. However, the underlying mechanisms involved in tumor formation are not well clarified. Interference of metal homeostasis may result in oxidative stress which represents an imbalance between production of free radicals and the system’s ability to readily detoxify reactive intermediates. This event consequently causes DNA damage, lipid peroxidation, protein modification, and possibly symptomatic effects for various diseases including cancer. This review discusses predominant modes of action and numerous molecular markers. Attention is paid to metal-induced generation of free radicals, the phenomenon of oxidative stress, damage to DNA, lipid, and proteins, responsive signal transduction pathways with major roles in cell growth and development, and roles of antioxidant enzymatic and DNA repair systems. Interaction of non-enzymatic antioxidants (carotenoids, flavonoids, glutathione, selenium, vitamin C, vitamin E, and others) with cellular oxidative stress markers (catalase, glutathione peroxidase, and superoxide dismutase) as well as certain regulatory factors, including AP-1, NF-κB, Ref-1, and p53 is also reviewed. Dysregulation of protective pathways, including cellular antioxidant network against free radicals as well as DNA repair deficiency is related to oncogenic stimulation. These observations provide evidence that emerging oxidative stress-responsive regulatory factors and DNA repair proteins are putative predictive factors for tumor initiation and progression.

## 1. General Features of Carcinogenic Metal Compounds

Metal compounds are found throughout the environment. Industrial applications contribute significantly to human metal exposure. Some metals, including arsenic, cadmium, chromium, cobalt, lead, mercury, and nickel have been classified as human carcinogens or considered to be human carcinogens by the International Agency for Research on Cancer and by the German MAK Commission. Their carcinogenic potentials are predominantly dependent on oxidation state, solubility, and complex form. Physicochemical properties control uptake, intracellular transport and distribution, and bioavailability [1–3]. Toxic metal ions, with similar properties to essential ions (e.g., charge and size), possibly compete with essential ions for biological binding sites, leading to perturbation of biomolecular structure and function as well as a disturbance in metal homeostasis [1–3]. Exposure to toxic metals is closely associated with the formation of free radicals, directly or indirectly, in living organisms [4–7]. The cumulative generation of free radicals, such as reactive oxygen species (ROS) and reactive nitrogen species (RNS), is termed oxidative stress and induces a cellular redox imbalance, which is linked to cancer incidence [8–10]. The common mode of action for metal-induced carcinogenicity is summarized as: (1) Induction of oxidative stress and damage to cellular components, particularly DNA; (2) interference with DNA repair systems, resulting in genomic instability; and (3) interruption of cell growth and proliferation via signaling pathways and dysregulation of oncogenes or tumor suppressor genes [1]. These possible common mechanisms of metal-induced carcinogenicity with unique discrepancies regarding to specific metals are discussed in more detail.

## 2. Overall Mechanisms of Metal-Induced Genotoxicity and Carcinogenicity

**Figure 1:**
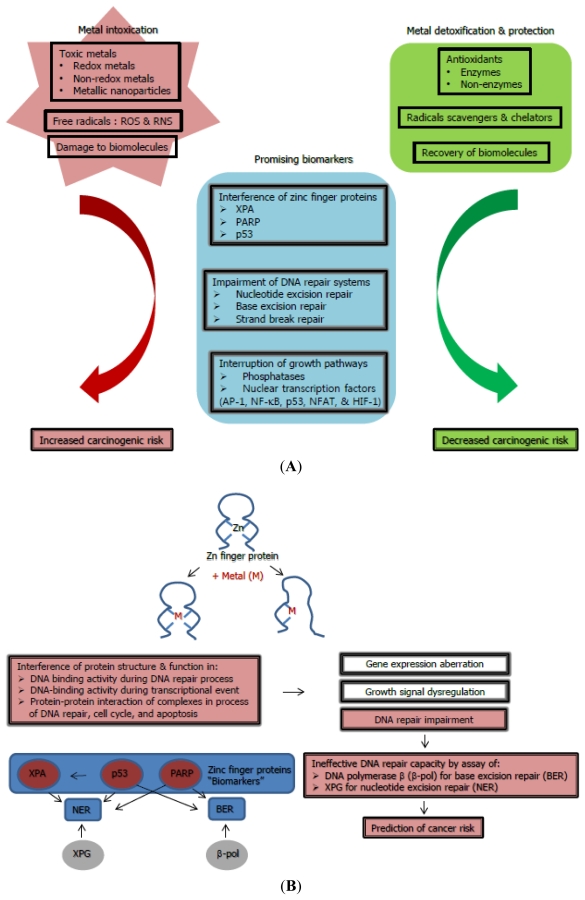
Scheme showing (**A**) main mechanisms and (**B**) plausible biomarkers with the proposed means of risk prediction for metal-mediated carcinogenicity.

Common mechanisms of various carcinogenic metals that induce oxidative stress, impair DNA repair systems, and interrupt signaling pathways are related to cell proliferation and account for the majority of metal-induced carcinogenicity (Figure 1) [1]. Unique mechanisms of specific carcinogenic metals cannot be excluded: such as substitution for inorganic phosphate in oxidative phosphorylation pathways by arsenic; disruption of cell-cell adhesion by cadmium; direct DNA binding of trivalent chromium; and interference with DNA methylation and histone acetylation by nickel.

### 2.1. Induction of Oxidative Stress, a Causative Source for Metal-Toxicity

Induction of oxidative stress is a remarkable phenomenon to explain metal-induced genotoxicity and mutagenicity. Several carcinogenic metals such as arsenic, cobalt, chromium, lead, mercury, and nickel induce redox reactions in living systems. These metals induce the production of ROS (e.g., hydroxyl peroxide and superoxide radicals) and RNS (e.g., nitric oxide, peroxynitrite and S-nitrosothiols) in both *in vivo* and *in vitro* systems. The generation of hydroxyl radicals has been mostly constituted through Fenton- and Haber-Weiss-type reactions. These radicals have rendered oxidative damage to DNA, proteins, and lipids. Redox-inert metal cadmium is unable to perform redox reactions in biological systems. However, it is able to stimulate oxidative stress as it inhibits antioxidant enzymes (e.g., catalase, glutathione peroxidase, glutathione reductase, and superoxide dismutase) through the interaction with their thiol groups, both *in vivo* and *in vitro*. Cadmium is also capable of replacing copper and iron in various cytoplasmic and membrane proteins (e.g., ferritin, apoferritin), leading to an increase in the amount of unbound or poorly chelated copper and iron ions inducing oxidative stress via Fenton reactions [11,12]. Cadmium treatment resulted in a significant number of cells with DNA single-strand breaks and cellular DNA damage [13,14]. Indeed, an animal system administrated with Cd(II) has transient oxidative damage [15,16]. Interestingly, a reversal effect of cobalt on free radical generation has been observed [17]. Intake of cobalt significantly suppresses the formation of free radical as well as oxidation of lipids and proteins. Other than direct DNA damage, ROS at low concentrations functions as a mitogenic signal to activate redox-sensitive transcription factors [1,18]. Chronic toxicity from persistent exposure to toxic metals leads to their accumulation in living system and becomes a health public concern [19–21]. Exposure to metallic compounds with nanoscalesizes, so called nanoparticles, such as cobalt and nickel has led to cytotoxic effect in concentration-dependent manner *in vitro* [22]. A number of studies have been conducted to investigate adverse effect of various metallic nanoparticles or nanomaterials on induction of free radicals as well as their modes of action both *in vitro* and *in vivo* [23–27]. These findings indicate several types of DNA damage, including generation of micronuclei, formation of DNA adduct (8-hydroxy-2-deoxyguanosine), and chromosomal aberrations. From, the results of nanoparticles-mediated expression analysis at both mRNA and protein also reveal extensive disruption of particular signaling pathways involving apoptosis, cell cycle control, embryogenesis, growth, and inflammation [27–31].

Therefore, oxidative stress might not only facilitate tumor initiation by mutagenesis but also deplete the activities of cellular antioxidant enzymes through the interactions with their thiol groups and dysregulate cell growth and proliferation, leading to tumor promotion. This event is predominantly dependent on the degree and duration of persistent exposure to carcinogenic metals. Oxidative stress phenomenon represent an interesting view of metal-induced carcinogenicity between relatively low doses of metals that are capable of inducing tumor initiation and highly cytotoxic doses of metals that elicit free radicals and damage to biomolecules. Hence, it is apparent that oxidative stress is not the sole causative factor for metal-initiated carcinogenesis but is still considered as potential contributor to malignant transformation.

### 2.2. Impairment of DNA Repair Systems and Involvement in Carcinogenesis

DNA molecules are continuously damaged by environmental stimuli (e.g., UV, chemical toxicants, and biological toxins) and endogenous factors formed during oxidative metabolism. Therefore, several endogenous DNA repair systems operate continuously with partial overlapping functions. These mainly include base excision repair (BER)/single strand break repair, nucleotide excision repair (NER), base mismatch repair, and recombinational (double strand break) repair. Most carcinogenic metals, except Cr(VI), are weak mutagens in mammalian cells and are often comutagenic due to the acceleration of mutagenicity of other genotoxic agents. A closer look reveals conflicting phenomenon under the aspect of DNA repair impairment in metal-mediated carcinogenesis: the presence of oxidative damage evoked by redox-inert metal like cadmium; discrepancies between low mutagenicity and high carcinogenicity for nickel compounds; and synergistic effects of coexposure to non-carcinogenic chemical such as polyaromatic hydrocarbons and cobalt [32]. Indeed, recent research has reported that some carcinogenic metals at low concentrations are able to inhibit repair of DNA damage generated by both endogenous and exogenous factors [1]. Increasing evidence has clarified that DNA repair processes are susceptible to carcinogenic metals, including As(III)/As(V), Cd(II), Cr(VI), Ni(II) as well as Hg(II) and Pb(II). Individual metals also inhibit selectively with repair systems at different steps. In the NER system, Cd(II) and Ni(II) interfere with the recognition of DNA lesions whereas Co(II) impairs the incision as well as the polymerization step [32–34]. As(III) inhibits the incision step at low concentrations and the ligation step at higher concentrations [34]. These three metals Cd(II), Hg(II), and Pb(II)also decrease the incision step [32,35]. A very recent report has documented that assembly and disassembly of the NER machinery are disturbed by water soluble Cd(II), as evidence of disassembly inhibition of XPA and XPC, principle components in the global genome NER [36]. Metalloids such as arsenic in particulate form, methylated arsenites and arsenates, are capable of inhibiting BER, NER, and strand break repair *in vitro* [37–39]. Chromate diminishes NER and synergistically augments mutagenicity of benzo[*a*]pyrene in mammalian cells [40]. A polymorphism of OGG1 enzyme involved in the BER process has shown repair susceptibility to chromate in human populations [41].

Inherited or acquired deficiencies in such repair systems can initiate to malignant growth. Genetic defects and polymorphisms in genes of the DNA repair components (e.g., ERCC1, MGMT, MLH1, MSH2, MSH6, and XRCC4) are strongly associated with human cancer [42,43]. Consecutive disturbance of repair and persistent DNA damage give rise to genomic instability, possibly allowing for aberrant cell proliferation and/or imperfect apoptosis.

### 2.3. Interruption of Cell Growth Signaling and Its Promotion of Carcinogenesis

Tumor development is likely attributable to dysregulation of cell growth and differentiation. Carcinogenic metals may affect cell growth by mechanisms such as changes in expression of growth-related factors and inactivation of growth regulation. Some metals promote several pathways, such as the mitogen-activated protein kinase (MAPK) pathways. This involves activation of nuclear transcription factors (AP-1, NF-κB, p53, NFAT, and HIF-1) which govern the expression of cytoprotective genes in relevance to DNA repair, immune response, cell cycle arrest, and apoptosis. Toxic metals and ROS probably interact with thiol groups in many types of phosphatases, particularly serine/threonine-, phosphotyrosine- and phospholipid-phosphatases, which are oxidized to form disulfide bonds [44,45]. This leads to protein conformational changes which in turn up-regulate various signaling cascades and result in the activation of particular redox-regulated transcription factors as mentioned above.

Nuclear transcription factor AP-1 has a vital role in cell growth and apoptosis [46]. AP-1 activity is induced by the JNK and p38 MAPK cascades in response to particular metals, hydrogen peroxide, cytokines, and other stressors [47]. Nuclear factor NF-κB has important functions in several processes, notably the inflammatory response, cell transformation, and cell survival [48,49]. Activation of NF-κB has been associated with carcinogenesis by external stimuli such as toxic metals, UV, and benzo[*a*]pyrene [48,49]. The influence of metals and ROS on NF-κB activation has been supported by the finding that the activation by several stimuli is often blocked by antioxidants, including thiols and vitamin E [50,51]. Administration of antioxidant *N*-acetyl-l-cysteine (NAC) significantly inhibits ROS production as well as NF-κB-, p38 MAPK-, and protein kinase *C*-mediated signaling pathways, resulting in abolished inflammation in rats treated with nanoparticles [24]. p53 gene mutations are linked to the majority of human cancers [52]. p53 mutation can occur by environmental carcinogens such as nickel, cigarette smoke, and UV irradiation [53,54]. Mechanisms of p53 activation in response to carcinogenic metals have determined in multiple ways [55,56]. The nuclear factor of activated T cells (NFAT) controls cytokine production, muscle growth and differentiation, and angiogenesis [57,58]. Previous studies have determined that various metals such as nickel increase intracellular calcium, representing a plausible mode of action for metal-activated NFAT [59]. Certain metals activate NFAT not only via a calcium-dependent pathway but also through formation of hydrogen peroxide [60]. Hypoxia-induced factor HIF-1 controls precise oxygen homeostasis by modulating expression of several cancer-related genes, including heme oxygenase1 and vascular endothelial growth factor [61]. The carcinogenic metals such as nickel or chromium and hydrogen peroxide have been known to activate HIF-1 [60,62]. *In vitro* study has reported that nickel activates the HIF-1 based on the substitution of iron in the oxygen carrier by nickel, which leads to permanent hypoxia; thus activating HIF1 [60].

Indeed, epigenetic mechanisms such as hypo- or hyper-methylation of DNA or altered histone acetylation might lead to changes in gene expression patterns. Alteration of gene regulation by carcinogenic metals in a persistent manner is linked to tumor manifestations. Some carcinogenic metals inhibit tumor suppressor p53 and/or decrease the expression of tumor suppressor genes (e.g., p16 and p53) as well as senescence genes. Therefore, metals might enhance cell proliferation by inhibiting apoptotic processes and allow for cell adaptation to metal-toxicity. Nickel alters normal growth control by distinct epigenetic mechanisms [63]. Recent studies have been conducted in mammalian cells in which nickel compounds enhance methylation of cytosine bases and reduce expression of tumor suppressor genes, resulting in accelerated cell proliferation. In nickel-induced tumors, DNA hypermethylation has been detected with decreased expression of the tumor suppressor genes p16 and Fhit. As a second epigenetic mechanism, nickel compounds have been observed to inhibit acetylation of several histones followed by chromatin condensation *in vitro*, probably by the binding of nickel ions to histone proteins. Because histone acetylation facilitates the accessibility of transcription factors to DNA, inhibition of histone acetylation apparently contributes to silencing of telomeric genes.

## 3. Revisiting Potential Biomarkers for Metal-Genotoxicity and Carcinogenicity: Interference of Protein-Protein Interactions with Zinc Finger Proteins

Approximately 10% of genesencode zinc finger proteins in the human genome with diverse ranges of functions, including DNA recognition and repair, RNA packaging, transcriptional activation, regulation of apoptosis, and protein folding and assembly [64–67]. Within zinc finger structures in their DNA-binding motifs, zinc is complexed to four cysteines (Cys) and/or histidines (His), allowing for proper folding of different structural domains and facilitating DNA-protein as well as protein-protein interactions [68,69]. Current data on interference with zinc finger DNA repair proteins by toxic metals will be summarized.

Indeed, interactions with zinc finger proteins, notably DNA repair proteins, transcription factors, and tumor suppressors, are thought to be more relevant for metal-mediated carcinogenesis rather than direct binding to DNA [1]. Possible mechanisms for zinc finger-interference by carcinogenic metals include isostructural substitution, replacement with altered geometry, mixed complex formation, and catalysis of thiol oxidation [69]. These modes related to metal carcinogenesis are dedicated to the altered gene expression [70]. Definite zinc finger proteins might be regarded as predictive direct biomarkers for the initiation of cancer (Figure 1).

### 3.1. Mammalian DNA Repair Protein XPA

Xerodermapigmentosum A (XPA) is a prominent protein, containing a single Cys4 zinc finger domain, for DNA lesion recognition in NER pathway [71,72]. NER is one of the most versatile repair pathways against various bulky DNA lesions, induced by UV light, environmental carcinogens, and particular anticancer agents. In the initial step, XPA is a key component in the assembly of the pre-incision complex by recruiting other proteins to the damaged DNA site. These include excision repair cross complementing protein 1, transcription factor IIH, and replication protein A. XPA binds specifically to DNA lesions induced by UVC, benzo[a]pyrene, or *cis*-platinum [73–75]. XPA contains a single zinc finger motif, as part of the minimal DNA-binding domain, in which four Cysresidues arecomplexed with zinc. Replacing of these individual Cysleads to a dramatic loss in NER activity [71]. Using a gel mobility shift assay, systematic studies focusing onmetal-inhibited XPA binding to a UV-irradiated oligonucleotide have shown that DNA binding activity is diminished by adding Cd(II), Co(II), and Ni(II) whereas it has not been affected by As(III), Hg(II), or Pb(II) [76] Simultaneous treatment with Zn(II) effectively prevents XPA inhibition by Cd(II), Co(II), and Ni(II) [76]. Parallel experiments with a bacterial form amidopyrimidine-DNA glycosylase (Fpg), a well-studied zinc finger protein responsible for BER, have revealed susceptibilities to Cd(II) and Hg(II)but no inhibitory effect of the other tested metals [76]. This reflects that individual zinc finger proteins have unique sensitivity to different toxic metals. Further molecular studies have been conducted using a structural model of the 37-peptide XPA zinc finger motif (XPAzf), allowing for competitive analysis of Zn(II) with Cd(II), Co(II), and Ni(II) in a quantitative manner [77,78]. In the presence of Ni(II), it has been found to promote XPAzf oxidation, resulting in the loss of Zn(II). This might be due to alterations in the tetrahedral geometry of the metal site and irreversible formation of intramolecular disulfide bonds catalyzed by Ni(II). In the case of Co(II), it demonstrates less effectiveness for Zn(II) substitution, relative to Ni(II). At excessive Co(II) concentrations, it has been observed to indirectly induce strong oxidation of XPAzf. To study of Cd(II), it is possible to substitute Zn(II) in quantitative fashion and consequently distort the peptide structure without Cd(II)-mediated oxidation of thiol groups, due to its very high binding affinity. These three metals are capable of interfering with XPA in different ways. Recent research has reported that soluble cadmium chloride obviously disturbs disassembly of XPA and XPC a key initiator in the global genome NER [36]. Collectively, other mechanisms of the exacerbated DNA-protein interactions via interference in its zinc finger motif by carcinogenic metals remain to be elucidated.

### 3.2. Poly (ADP-Ribose)Polymerase(PARP)

DNA strand break repair protein PARP possesses two separateCys3His1-type zinc finger domains with the main role of detecting and signaling DNA strand breaks for enzymatic machinery involved in BER [79]. Following DNA strand breakage, PARP catalyzes the addition of long chains of poly(ADP-ribose)polymers to target proteins participating in chromatin architecture and DNA metabolism [80–82]. This modification step seems to be obligatory for detecting and/or recognizing nicked DNA [82,83]. Under mild to moderate genotoxic stimuli, PARP proceeds with the DNA repair process through cell cycle arrest and subsequent interaction with DNA repair enzymes [80,82]. PARP may be hyperactivated by severe DNA damage which eventually stimulates the apoptotic process [82,84]. Furthermore, PARP likely has a profound role in anticancer agent-induced and spontaneous apoptosis; however, it is not yet fully clarified [85]. A very recent investigation evaluated inhibitory effects of anticancer metal complexes on PARP activity derived from human cancer cells, indicating a strong link between PARP inhibition and binding ability of these complexes to the zinc finger motif by zinc competition [82]. These data support the concept that zinc displacement with other metals in the zinc finger motif yields a reduction in its activity. This result emphasizes that PARP is promising mediator involved in drug resistance to cancer cell chemotherapies. PARP activity is reduced in a human lymphoma cell line by As(III) [86]. Similar experiments have demonstrated that hydrogen peroxide-induced PARP activity in HeLa cells is selectively inhibited by As(II), Co(II), Cd(II), and Ni(II) but not by Pb(II) or Hg(II) [68]. However, further molecular studies based on inhibition via interactions with the zinc finger motif are necessary.

### 3.3. Tumor Suppressor Protein p53

The p53 protein with Cys3His1-typed zinc finger domain has an important role in DNA repair through the NER and genomic stability [87]. p53 controls a number of key events to induce either DNA repair processing, cell cycle arrest, or apoptosis via coordinated pathways, depending on the physiological state and cell type [87]. Indeed, p53 is activated by multiple forms of stress signals, including DNA damage. p53 regulates the transcription of several downstream genes including XPA, via its binding to specific response elements, which prevents damaged cells from dividing, leading to repair of damaged DNA, or ultimately to cells with severe damage by apoptosis. In addition, p53 directly interact with particular proteins involved in DNA repair, replication, and transcription [88]. Based on its key biochemical property, sequence-specific DNA binding is dependent on metal and redox regulation. p53-DNA binding is mediated by tetrahedral co-ordination of zinc with three Cys and one Hys, suggesting that zinc is required for proper folding of p53 into its native conformation and consequent functionality [89]. Selenium compounds, as redox stimulators, may facilitate p53-specific DNA binding as well as p53-mediated DNA repair through a redox regulation at definite Cys residues, in the presence of DNA damage [90–95]. Co(II) and Ni(II) impair p53-DNA binding capacity and inhibit cell cycle arrest [96], due to the disruption of p53 native conformation. Water-soluble cadmium chloride and particulate cadmium oxide compounds also altered p53 conformation at the zinc finger motif in human cells [36,97].

Taken together, zinc finger motifs participate in protein–DNA and protein-protein interactions in several groups of proteins, including those involved in DNA repair, transcription, and tumor suppression. Definite carcinogenic metals, such as arsenic, cadmium, cobalt, lead, and nickel differentially inhibit these zinc finger proteins, leading to distorted zinc finger domains and consequent dysfunction of the proteins. Therefore, this reactivity could be considered as a plausible molecular mechanism in carcinogenesis. Indeed, very limited information has established reference concentration (RfC) or reference dose (RfD) for the exposure of individual carcinogenic metals, indicating no confident association between metal levels and their carcinogenicities in humans. Besides direct measurement of metal levels in human specimens, the use of these zinc finger proteins as genetic factors could predict risk of carcinogenesis with a higher specificity based on changes in their binding constants or stability constants when complexed with either Zn(II) or thiol-typed antioxidants (e.g., glutathione andthioredoxin) [16,66]. Alternatively, assessing DNA repair capacity via assays of particular enzymes (DNA polymerase β “β pol” and XPG or ERCC5) involved in definite zinc finger protein-modulated repair pathways, with the use of protein extracts from human tissues or cells, might be useful for partially evaluating cancer risk [98,99].

## 4. Enhancement of Antioxidant Defense Systems Responsible for Reducing Metal-Induced Carcinogenicity

The interactions of various carcinogenic metals with biological components are quite complex. The cellular components in antioxidant defenses are vitalas they scavenge and balance prooxidants, ROS and RNS, which are attributable to the activities of antioxidant enzymes as well as the action of non-enzymatic antioxidants (Figure 1), thus providing maximal protection fat biological sites. The most efficient enzymatic antioxidants are comprised of catalase, glutathione peroxidase, and superoxide dismutase [10,100]. Non-enzymatic antioxidants include vitamin C, vitamin E, thiol antioxidants (glutathione, thioredoxin, and lipoic acid), natural flavonoids, melatonin, and selenium [101]. Some antioxidants such as vitamin C act in a hydrophilic phase, some such as vitamin E in a hydrophobic phase, and others such as α-lipoic acid act in both phases. Indeed, the capacity to regenerate one antioxidant by another, called an antioxidant network is driven by redox potentials [102]. There is correlation between enhanced ROS levels and abolished activities of both enzymatic and non-enzymatic antioxidants in tumor cells.

### 4.1. Enzymatic Antioxidants and Their Physiological Response to Metallotoxicity

Several studies have documented altered levels of reduced glutathione and glutathione peroxidase in animals following arsenic exposure [4,103,104]. In the case of cadmium, changes in antioxidant enzymes activities (e.g., catalase, glutathione peroxidase, glutathione reductase, glutathione-*S*-transferase, and Cu, Zn-superoxide dismutase) have been determined in rats [105,106]. Lead exposure results in alterations of oxidative stress markers (catalase, superoxide dismutase and glutathione peroxidase, and glutathione reductase) as well as the level of reduced glutathione in animal models [107,108].

Although adding antioxidant enzymes such as catalase and superoxide dismutase (75 and 150 μg/mL, respectively) does not appear to protect lymphocytes against organic mercury-induced genotoxicity *in vitro* [109,110], epidemiological observations have revealed that the activity of these enzymes changes in exposed populations with resulting genotoxic alterations [109,111]. These data emphasize that chronic exposure to relatively low levels of mercury may inhibit antioxidant enzymatic activity due to persistent oxidative stress [109,111]. This phenomenon might represent an important peripheral target for mercury toxicity in exposed populations.

### 4.2. Non-Enzymatic Antioxidants and Their Melioration towards Carcinogenesis

Supplementation with vitamin C and/or vitamin E is protective against cadmium intoxication as shown by the decrease in ROS level in rat testicular tissues. Dietary uptake of a combination of these vitamins restores normal testicular function in cadmium-treated rats [112]. Effective antioxidants and free radical scavengers such as melatonin, methyl gallate, and quercetin also have cytoprotective effect against cadmium toxicity by reducing lipid peroxidation and maintaining physiological homeostasis [113–115]. Inhaled cobalt particles interact primarily with surfactants and antioxidants on the lung surface [116]. Reduced glutathione, a ROS scavenger, acts as one of the first lines of defense against lung injury due to formation of excessive ROS. The extent of the reduction in thiol concentration is correlated with the amount of dust and consequent surface area exposed. Co(II) exposure also results in the depletion of intracellular ascorbate [117]. Intriguingly, the influx of ascorbate is inhibited by cobalt whereas the efflux is metal-independent process. Both ascorbate and reduced glutathione can scavenge superoxide and hydroxyl radicals, initiated by cobalt [116,118]. Additionally, reduced glutathione and Cysresidues in proteins also have a prominent role in redox regulation in response to cobalt in the form of a cobalt/tungsten (Co/WC) mixture [116,118].

Previous reports suggest that ascorbate is a primary reducer of Cr(VI) in cells [119–121]. However, ascorbate has dual opposing roles in Cr(VI) intoxication, as a protective-antioxidant outside and a prooxidant inside of cells. The ascorbate-initiated reduction of Cr(VI) inside cells cause high amounts of chromium-DNA adducts, allowing for DNA mutation [120–122]. In addition, Cr(VI)is also reduced through non-enzymatic reactions with Cys and glutathione. The primary reductant of Cr(VI)in mitochondria seems to be NAD(P)H, resulting in stable Cr(III) with relatively higher DNA affinity than that of Cr(VI) [2,122].

The majority of lead intoxication is related to glutathione metabolism [4,123]. Lead exposure in animal models alters the reduced glutathione level [107,108]. Glutathione is an important substrate, which impacts the action of several drugs and toxins via its conjugation in the liver. An increase in the incidence of hypertension has been observed following lead exposure in humans, possibly due to significant effect of RNS such as nitric oxide [124]. Antioxidants can be utilized to inhibit the availability of nitric oxide. Administration of vitamin E (5000 IU/kg) and vitamin C (3 mmol/L of drinking water) to hypertensive rats with suppressed glutathione formation abolish shypertension. Zinc supplementation in lead-treated animals restores superoxide dismutase levels [125], suggesting that zinc acts as an antioxidant and a plausible chelating agent toward lead toxicity. Administration of selenium prior to lead exposure is plays a protective effect in animals [126]. Selenium elevates reduced glutathione, glutathione peroxidase, and superoxide dismutase levels in kidney and liver tissues as well as reduced glutathione. Selenium generates a stable lead-selenium complex, implying protective effect against lead toxicity. Alpha-lipoic acid is an effective antioxidant with chelating properties. In response to lead exposure, alpha-lipoic acid suppresses the deleterious effect of lead on glutathione and oxidative stress markers in liver and kidney tissues [127].

In studies of mercury-induced toxicity, increased glutathione levels may protect cells by exerting antioxidant activity and chelating mercury [109,128], suggesting that glutathione acts as a major line of cell defense against mercury toxicity. Previous data also suggest that high levels of intracellular glutathione may contribute to neuro protection upon exposure to mercury compounds [129]. *In vivo* research has demonstrated that glutathione levels are also higher in human populations that consume methylmercury-contaminated fish (levels of mercury content in hair of 12–15 μg/g) [111]. Furthermore, a direct positive relationship between glutathione and mercury levels has been found in the blood. Antioxidant substances such as ascorbate also exhibit their protective action against mercury genotoxicity *in vitro* by preventing sister chromatid exchanges and abnormal mitosis [130]. Interestingly, a substantial inverse correlation was observed between mercury levels in blood and consumption of tropical fruits (particularly vitamin C-rich oranges) in mercury-exposed populations [131]. This reflects another aspect of the protective role of the antioxidant compounds.

Nevertheless, a meta-analysis of randomized controlled trials has revealed inconsistent findings between experimental studies (both *in vitro* and *in vivo*) and human clinical trials with regard to the association between antioxidant supplements and the risk of carcinogenesis, suggesting that experimental studies showing the effects of antioxidant substances cannot be directly applied to humans [132] because these substances might exert adverse properties or promote carcinogenesis under clinical circumstances. Currently, no clinical evidence supports the use of antioxidant supplements for primary and secondary prevention of cancer. Although many populations consume antioxidant substances to improve their health and prevent cancer, the potential effects (either beneficial or deleterious) of antioxidant supplements on human health, particularly in relation to cancer risk, must be emphasized.

## 5. Conclusion

The relevance between metal-induced interference and carcinogenesis in living systems has received increasing attention. Understanding the mechanisms underlying toxicity initiated by diverse carcinogenic metals and metallic nanoparticles is of great concern. Little is known about toxicity and guidelines based on metallic nanoparticles whereas their trend of use has been increasing. Chronic exposure to toxic metals via diverse routes into living bodies can cause accumulation and dangerous illness [21,133–136]. Among children, toxic metal biomonitoring in physiological tissues such as blood and urine may provide adequate assessments to prevent such illness/suffering, leading to severe mental retardation [19,21]. Metal carcinogenicity occurs through complex mechanisms and oxidative assault, cellular redox homeostasis, DNA repair, and particular signal transduction pathways are thought to be interconnected. The interference of toxic metals with zinc finger proteins, notably DNA repair proteins, is likely to be more relevant for metal-mediated carcinogenesis than for direct interactions with DNA [1]. The relevant issue of zinc finger proteins functioning in DNA repair systems is also supported by epidemiological observations that a large number of human populations with inadequate dietary zinc, even in developed countries, have augmented cancer incidence [16,137]. Zinc deficiency probably results in enhanced displacement and lowered restoration of zinc finger motifs which are present in zinc finger proteins (e.g., DNA repair enzymes, nuclear transcription factors, and tumor suppressors). Hence, a broad spectrum of these zinc finger proteins might be regarded as promising biomarkers for risk assessment of environmental and occupational exposure to definite carcinogenic metals. Identifying novel markers or a collection of specific markers will be further required for developing a test so that the defects will be detected as early as possible. These studies will be helpful for further developing a robust risk evaluation and improving public health protection. Significant action to reduce cancer incidence relevant to oxidative stress seems to be counteracted by non-enzymatic antioxidants cooperating with cellular antioxidant enzymes. The most important action of cancer prevention is to minimize exposure to oxidative stressors, particularly exogenous sources.

## References

[1] Beyersmann D., Hartwig A. (2008). Carcinogenic metal compounds: Recent insight into molecular and cellular mechanisms. Arch. Toxicol.

[2] Hartwig A. (2001). Zinc finger proteins as potential targets for toxic metal ions: Differential effects on structure and function. Antioxid. Redox Signal.

[3] Nieboer E., Fletcher G.G., Thomassen Y. (1999). Relevance of reactivity determinants to exposure assessment and biological monitoring of the elements. J. Environ. Monit.

[4] Jomova K., Valko M. (2011). Advances in metal-induced oxidative stress and human disease. Toxicology.

[5] Matés J.M., Pérez-Gómez C., de Castro I.N., Asenjo M., Márquez J. (2002). Glutamine and its relationship with intracellular redox status, oxidative stress and cell proliferation/death. Int. J. Biochem. Cell Biol.

[6] Matés J.M., Segura J.A., Alonso F.J., Márquez J. (2008). Intracellular redox status and oxidative stress: Implications for cell proliferation, apoptosis, and carcinogenesis. Arch. Toxicol.

[7] Rahman K. (2007). Studies on free radicals, antioxidants, and co-factors. Clin. Interv. Aging.

[8] Poli G., Leonarduzzi G., Biasi F., Chiarpotto E. (2004). Oxidative stress and cell signalling. Curr. Med. Chem.

[9] Valko M., Izakovic M., Mazur M., Rhodes C.J., Telser J. (2004). Role of oxygen radicals in DNA damage and cancer incidence. Mol. Cell. Biochem.

[10] Valko M., Rhodes C.J., Moncol J., Izakovic M., Mazur M. (2006). Free radicals, metals and antioxidants in oxidative stress-induced cancer. Chem. Biol. Interact.

[11] Price D.J., Joshi J.G. (1983). Ferritin. Binding of beryllium and other divalent metal ions. J. Biol. Chem.

[12] Casalino E., Sblano C., Landriscina C. (1997). Enzyme activity alteration by cadmium administration to rats: The possibility of iron involvement in lipid peroxidation. Arch. Biochem. Biophys.

[13] Park J.Y., Seo Y.R. (2011). The protective role of Nrf2 in cadmium-induced DNA damage. Mol. Cell. Toxicol.

[14] Yang J.M., Arnush M., Chen Q.Y., Wu X.D., Pang B., Jiang X.Z. (2003). Cadmium-induced damage to primary cultures of rat Leydig cells. Reprod. Toxicol.

[15] Qu W., Diwan B.A., Reece J.M., Bortner C.D., Pi J., Liu J., Waalkes M.P. (2005). Cadmium-induced malignant transformation in rat liver cells: Role of aberrant oncogene expression and minimal role of oxidative stress. Int. J. Cancer.

[16] Witkiewicz-Kucharczyk A., Bal W. (2006). Damage of zinc fingers in DNA repair proteins, a novel molecular mechanism in carcinogenesis. Toxicol. Lett.

[17] Shukla D., Saxena S., Jayamurthy P., Sairam M., Singh M., Jain S.K., Bansal A., Ilavazaghan G. (2009). Hypoxic preconditioning with cobalt attenuates hypobaric hypoxia-induced oxidative damage in rat lungs. High Alt. Med. Biol.

[18] Genestra M. (2007). Oxyl radicals, redox-sensitive signallingcascades and antioxidants. Cell. Signal.

[19] Kim D.-S., Yu S.-D., Lee E.-H. (2010). Effects of blood lead concentration on intelligence and personality in school children. Mol. Cell. Toxicol.

[20] Clarkson T.W., Magos L. (2006). The toxicology of mercury and its chemical compounds. Crit. Rev. Toxicol.

[21] Kim D.S., Kim J.H., Yang W.-H., Moon J.S., Son B.S. (2010). Biomonitoring of urinary mercury in Korean school children. Mol. Cell. Toxicol.

[22] Peters K., Unger R.E., Gatti A.M., Sabbioni E., Tsaryk R., Kirkpatrick C.J. (2007). Metallic nanoparticles exhibit paradoxical effects on oxidative stress and pro-inflammatory response in endothelial cells *in vitro*. Int. J. Immunopathol. Pharmacol.

[23] Khalil W.K., Girgis E., Emam A.N., Mohamed M.B., Rao K.V. (2011). Genotoxicity evaluation of nanomaterials: DNA damage, micronuclei, and 8-hydroxy-2-deoxyguanosine induced by magnetic doped CdSe quantum dots in male mice. Chem. Res. Toxicol.

[24] Kim J.-H., Jang A.-S., Shin E.K., Kang C.-M., Seok J., Lee E.H., Kim M.O., Park S.W., Uh S.T., Park C.-S. (2010). Particle-induced expression of SF20/IL25 is mediated by reactive oxygen species and NF-κB in alveolar macrophages. Mol. Cell. Toxicol.

[25] Kim Y.-J., Yang S.I., Ryu J.-C. (2010). Cytotoxicity and genotoxicity of nano-silver in mammalian cell lines. Mol. Cell. Toxicol.

[26] Park H.-O., Yu M., Kang S.K., Yang S.I., Kim Y.-J. (2011). Comparison of cellular effects of titanium dioxide nanoparticles with different photocatalytic potential in human keratinocyte, HaCaT cells. Mol. Cell. Toxicol.

[27] Yeo M.-K., Kang M.-S. (2010). The effect of nano-scale Zn-doped TiO_2_ and pure TiO_2_ particles on *Hydra magnipapillata*. Mol. Cell. Toxicol.

[28] Jeon Y.-M., Park S.-K., Rhee S.-K., Lee M.-Y. (2010). Proteomic profiling of the differentially expressed proteins by TiO_2_ nanoparticles in mouse kidney. Mol. Cell. Toxicol.

[29] Yeo M.-K., Kim H.-E. (2010). Gene expression in zebrafish embryos following exposure to TiO_2_ nanoparticles. Mol. Cell. Toxicol.

[30] Yeo M.K., Park S.W. (2008). Exposing zebrafish to silver nanoparticles during caudal fin regeneration disrupts caudal fin growth and p53 signaling. Mol. Cell. Toxicol.

[31] Yeo M.K., Yoon J.W. (2009). Comparison of the effects of nano-silver antibacterial coating and silver ions on zebrafish embryogenesis. Mol. Cell. Toxicol.

[32] Hartwig A., Schwerdtle T. (2002). Interactions by carcinogenic metal compounds with DNA repair processes: Toxicological implications. Toxicol. Lett.

[33] Kasten U., Mullenders L.H., Hartwig A. (1997). Cobalt(II) inhibits the incision and the polymerization step of nucleotide excision repair in human fibroblasts. Mutat. Res.

[34] Hartwig A., Mullender L., Asmuβ M., Dally H., Hartmann M. (1998). Disruptions of DNA repair processes by carcinogenic metal compounds. Fresenius J. Anal. Chem.

[35] Calsoul P., Frit P., Bozzato C., Salles B. (1996). Negative interference of metal(II) ions with nucleotide excision repair in human cell-free extracts. Carcinogenesis.

[36] Schwerdtle T., Ebert F., Thuy C., Richter C., Mullenders L.H., Hartwig A. (2010). Genotoxicity of soluble and particulate cadmium compounds: Impact on oxidative DNA damage and nucleotide excision repair. Chem. Res. Toxicol.

[37] Hartwig A., Blessing H., Schwerdtle T., Walter I. (2003a). Modulation of DNA repair processes by arsenic and selenium compounds. Toxicology.

[38] Hartwig A., Pelzer A., Asmuss M., Bürkle A. (2003b). Very low concentrations of arsenite suppress poly(ADP-ribosyl)ation in mammalian cells. Int. J. Cancer.

[39] Schwerdtle T., Walter I., Hartwig A. (2003). Arsenite and its biomethylated metabolites interfere with the formation and repair of stable BPDE-induced DNA adducts in human cells and impair XPAzf and Fpg. DNA Repair.

[40] Hu W., Feng Z., Tang M. (2004). Chromium(VI) enhances (±)-*anti*-7β,8α-dihydroxy-9α,10α-epoxy-7,8,9,10-tetrahydrobenzo[*a*]pyrene-induced cytotoxicity and mutagenicity in mammalian cells through its inhibitory effect on nucleotide excision repair. Biochemistry.

[41] Lee A.J., Hodges N.J., Chipman J.K. (2005). Interindividual variability in response to sodium dichromate-induced oxidative DNA damage: Role of the Ser326Cys polymorphism in the DNA repair protein of 8-oxo-7,8-dihydro-2′-deoxyguanosine DNA glycosylase 1. Cancer Epidermiol. Biomark. Prev.

[42] Ford B.N., Ruttan C.C., Kyle V.L., Brackley M.E., Glickman B.W. (2000). Identification of single nucleotide polymorphisms in human DNA repair genes. Carcinogenesis.

[43] Nohmi T., Kim S.R., Yamada M. (2005). Modulation of oxidative mutagenesis and carcinogenesis by polymorphic forms of human DNA repair enzymes. Mutat. Res.

[44] Poli G., Leonarduzzi G., Biasi F., Chiarpotto E. (2004). Oxidative stress and cell signalling. Curr. Med. Chem.

[45] Thannickal V.J., Fanburg B.L. (2000). Reactive oxygen species in cell signaling. Am. J. Physiol. Lung Cell. Mol. Physiol.

[46] Whitmarsh A.J., Davis R.J. (1996). Transcription factor AP-1 regulations by mitogen-activated protein kinase signal transduction pathways. J. Mol. Med.

[47] Pinkus R., Weiner L.M., Daniel V. (1996). Role of oxidants and antioxidants in the induction of AP-1, NF-kappa B, and glutathione *S*-transferase gene expression. J. Biol. Chem.

[48] Amiri K.I., Richmond A. (2005). Role of nuclear factor-kappa B in melanoma. Cancer Metastasis Rev.

[49] Pande V., Ramos M.J. (2005). NF-kappa B in human disease: Current inhibitors and prospects for de novo structure based design of inhibitors. Curr. Med. Chem.

[50] Knight J.A. (2000). Free radicals, antioxidants, and the immune system. Ann. Clin. Lab. Sci.

[51] Hughes G., Murphy M.P., Ledgerwood E.C. (2005). Mitochondrial reactive oxygen species regulate the temporal activation of nuclear factor kappa B to modulate tumour necrosis factor-induced apoptosis: Evidence from mitochondria-targeted antioxidants. Biochem. J.

[52] Hollstein M., Sidransky D., Vogelstein B., Harris C.C. (1991). p53 mutations in human cancer. Science.

[53] Maehle L., Metcalf R.A., Ryberg D., Bennett W.P., Harris C.C., Haugen A. (1992). Altered p53 gene structure and expression in human epithelial cells after exposure to nickel. Cancer Res.

[54] Olivier M., Hussain S.P., de Fromentel C.C., Hainaut P., Harris C.C. (2004). TP53 mutation specta and load: A tool for generating hypotheses on the etiology of cancer. IARC Sci. Publ.

[55] Huang C., Ma W.Y., Li J., Dong Z. (1999). Arsenic induces apoptosis through a c-Jun NH_2_-terminal kinase-dependent, p53-independent pathway. Cancer Res.

[56] Wang S.W., Shi X.L. (2001). Mechanisms of Cr(VI)-induced p53 activation: The role of phosphorylation, mdm2 and ERK. Carcinogenesis.

[57] Jauliac S., Lopez-Rodriguez C., Shaw L.M., Brown L.F., Rao A., Toker A. (2002). The role of FAT transcription factors in integrin-mediated carcinoma invasion. Nat. Cell Biol.

[58] Rao A., Luo C., Hogan P.G. (1997). Transcription factors of the NFAT family: Regulation and function. Ann. Rev. Immunol.

[59] Chow C.W., Rincon M., Cavanagh J., Dickens M., Davis R.J. (1997). Nuclear accumulation of NFAT4 opposed by the JNK signal transduction pathway. Science.

[60] Leonard S.S., Harris G.K., Shi X.L. (2004). Metal-induced oxidative stress and signal transduction. Free Radic. Biol. Med.

[61] Semenza G.L. (2000). HIF-1: Mediator of physiological and pathophysiological responses to hypoxia. J. Appl. Physiol.

[62] Gao N., Jiang B.H., Leonard S.S., Corum L., Zhang Z., Roberts J.R., Antonini J., Zheng J.Z., Flynn D.C., Castranova V. (2002). p38 signaling-mediated hypoxia-inducible factor 1 alpha and vascular endothelial growth factor induction by Cr(VI) in DU145 human prostate carcinoma cells. J. Biol. Chem.

[63] Salnikow K., Zhitkovich A. (2008). Genetic and epigenetic mechanisms in metal carcinogenesis and cocarcinogenesis: Nickel, arsenic, and chromium. Chem. Res. Toxicol.

[64] Maret W., Li Y. (2009). Coordination dynamics of zinc in proteins. Chem. Rev.

[65] Parkin G. (2004). Synthetic analogues relevant to the structure and function of zinc enzymes. Chem. Rev.

[66] Quintal S.M., de Paula Q.A., Farrell N.P. (2011). Zinc finger proteins as templates for metal ion exchange and ligan reactivity. Chemical and biological consequences. Metallomics.

[67] de Paula Q.A., Mangrum J.B., Farrell N.P. (2009). Zinc finger proteins as templates for metal ion exchange: Substitution effects on the C-finger of HIV nucleocapsid NCp7 using M (chelate) species (M = Pt, Pd, Au). J. Inorg. Biochem.

[68] Hartwig A., Asmuss M., Blessing H., Hoffmann S., Jahnke G., Khandelwal S., Pelzer A., Bürkle A. (2002). Interference by toxic metal ions with zinc-dependent proteins involved in maintaining genomic stability. Food Chem. Toxicol.

[69] Hartwig A. (2001). Zinc finger proteins as potential targets for toxic metal ions: Differential effects on structure and function. Antioxid. Redox Signal.

[70] Sunderman F.W., Barber A.M. (1988). Finger-loops, oncogenes, and metals. Claude Passmore Brown Memorial Lecture. Ann. Clin. Lab. Sci..

[71] Miyamoto I., Miura N., Niwa H., Miyazaki J., Tanaka K. (1992). Mutational analysis of the structure and function of the xerodermapigmentosum group A complementing protein. J. Biol. Chem.

[72] Riedl T., Hanaoka F., Egly J.-M. (2003). The comings and goings of nucleotide excision repair factors on damaged DNA. EMBO J.

[73] Asahina H., Kuraoka I., Shirakawa M., Morita E.H., Miura N., Miyamoto I., Ohtsuka E., Okada Y., Tanaka K. (1994). The XPA protein a zinc metalloprotein with an ability to recognize various kinds of DNA damage. Mutat. Res.

[74] Jones C.J., Wood R.D. (1993). Preferential binding of the xerodermapigmentosum group A complementing protein to damaged DNA. Biochemistry.

[75] Robins P., Jones C.J., Biggerstaff M., Lindahl T., Wood R.D. (1991). Complementation of DNA repair in xerodermapigmentosum group A cell extracts by a protein with affinity for damaged DNA. EMBO J.

[76] Asmuss M., Mullenders L.H.F., Eker A., Hartwig A. (2000). Differential effects of toxic metal compounds on the activities of Fpg and XPA, two zinc finger proteins involved in DNA repair. Carcinogenesis.

[77] Bal W., Schwerdtle T., Hartwig A. (2003). Mechanism of nickel assault on the zinc finger of DNA repair protein XPA. Chem. Res. Toxicol.

[78] Kopera E., Schwerdtle T., Hartwig A., Bal W. (2004). Co(II) and Cd(II) substitute for Zn(II) in the zinc finger derived from the DNA repair protein XPA, demonstrating a variety of potential mechanisms of toxicity. Chem. Res. Toxicol.

[79] Gradwohl F., de Murcia J.M.M., Molinete M., Simonin F., Koken M., Hoeijmakers J.H., de Murcia G. (1990). The second zinc-finger domain of poly(ADP-ribose) polymerase determines specificity for single-stranded breaks in DNA. Proc. Natl. Acad. Sci. USA.

[80] Kim M.Y., Zhang T., Kraus W.L. (2005). Poly(ADP-ribosyl)ation by PARP-1: ‘PAR-laying’ NAD^(+)^ into a nuclear signal. Genes Dev.

[81] Homburg S., Visochek L., Moran N., Dantzer F., Priel E., Asculai E., Schwartz D., Rotter V., Dekel N., Cohen-Armon M. (2000). A fast signal-induced activation of poly(ADP-ribose) polymerase: A novel downstream target of phospholipase C. J. Cell Biol.

[82] Mendes F., Groessl M., Nazarov A.A., Tsybin Y.O., Sava G., Santos I., Dyson P.J., Casini A. (2011). Metal-based inhibition of poly(ADP-ribose) polymerase-the guardian angel of DNA. J. Med. Chem.

[83] de Murcia G., de Murcia J.M. (1994). Poly(ADP-ribose) polymerase-A molecular nick-sensor. Trends Biochem. Sci.

[84] Herceg Z., Wang Z.Q. (2001). Functions of poly(ADP-ribose) polymerase (PARP) in DNA repair, genomic integrity and cell death. Mutat. Res. Fundam. Mol. Mech. Mutagen.

[85] de Murcia G., Shall S. (2000). Poly(ADP-ribosylation) reactions: From DNA damage and stress signalling to cell death. Mutat. Res.

[86] Yager J.W., Wiencke J.K. (1997). Inhibition of poly(ADP-ribose) polymerase by arsenite. Mutat. Res.

[87] Hainaut P., Mann K. (2001). Zinc binding and redox control of p53 structure and function. Antioxid. Redox Signal.

[88] Hainaut P., Hollstein M. (1999). p53 and human cancer: The first ten thousand mutations. Adv. Cancer Res.

[89] Méplan C., Richard M.J., Hainaut P. (2000). Redox signalling and transition metals in the control of p53 pathway. Biochem. Pharmacol.

[90] Fischer J.L., Mihelc E.M., Pollok K.E., Smith M.L. (2007). Chemotherapeutic selectivity conferred by selenium: A role for p53-dependent DNA repair. Mol. Cancer Ther.

[91] Jung H.J., Seo Y.R. (2010). Current issues of selenium in cancer chemoprevention. Biofactors.

[92] Jung H.J., Lee J.H., Seo Y.R. (2009). Enhancement of methylmethanesulfonate-induced based excision repair in the presence of selenomethionine on p53-dependent pathway. J. Med. Food.

[93] Seo Y.R., Kelley M.R., Smith M.L. (2002). Selenomethionine regulation of p53 by a ref 1-dependent redox mechanism. Proc. Natl. Acad. Sci. USA.

[94] Seo Y.R., Sweeney C., Smith M.L. (2002). Selenomethionine induction of DNA repair response in human fibroblasts. Oncogene.

[95] Smith M.L., Lancia J.K., Mercer T.I., Ip C. (2004). Selenium compounds regulate p53 by common and distinctive mechanisms. Anti-Cancer Res.

[96] Palecek E., Brazdova M., Cemocka H., Vlk D., Brazda V., Vojtesek B. (1999). Effect of transition metals on binding of p53 protein to supercoiled DNA and to consensus sequence in DNA fragments. Oncogene.

[97] Meplan C., Mann K., Hainaut P. (1999). Cadmium induces conformational modifications of wild-type p53 and suppress p53 response to DNA damage in cultured cells. J. Biol. Chem.

[98] Cabelof D.C., Raffoul J.J., Yanamadala S., Ganir C., Guo Z.M., Heydari A.R. (2002). Attenuation of DNA polymerase-dependent base excision repair and increased DMS-induced mutagenicity in aged mice. Mutat. Res.

[99] Koedrith P., Seo Y.R. (2004). Development of quantitative DNA cleavage assay for XPG endonuclease activity using endogenous nuclear proteins in human cell lines. Oncol. Rep.

[100] Mates J.M., Perez-Gomez C., de Castro I.N. (1999). Antioxidant enzymes and human diseases. Clin. Biochem.

[101] McCall M.R., Frei B. (1999). Can antioxidant vitamins materially reduce oxidative damage in humans?. Free Radic. Biol. Med.

[102] Sies H., Stahl W., Sevanian A. (2005). Nutritional, dietary and post-prandial oxidative stress. J. Nutr.

[103] Halliwell B., Gutteridge J.M.C. (1990). Role of free radicals and catalytic metal-ions in human disease-an overview. Methods Enzymol.

[104] Maiti S., Chatterjee A.K. (2001). Effects on levels of glutathione and some related enzymes in tissues after an acute exposure in rats and their relationship to dietary protein deficiency. Arch. Toxicol.

[105] Novelli E.I.B., Marques S.F.G., Almeida J.A., Diniz Y.S., Faine L.A., Ribas B.O. (2000). Toxic mechanism of cadmium exposure on cardiac tissue. Toxic Subst. Mech.

[106] Ognjanovic B.I., Pavlovic S.Z., Maletic S.D., Zikic R.V., Stajn A.S., Radojicic R.M., Saicic Z.S., Petrovic V.M. (2003). Protective influence of vitamin E on antioxidant defense system in the blood of rats treated with cadmium. Physiol. Res.

[107] Ahamed M., Verma S., Kumar A., Siddiqui M.K.J. (2005). Environmental exposure to lead and its correlation with biochemical indices in children. Sci. Total Environ.

[108] Hoffman D.J., Heinz G.H., Sileo L., Audet D.J., Campbell J.K., Obrecht H.H. (2000). Developmental toxicity of lead-contaminated sediment in Canada geese (*Branta canadensis*). J. Toxicol. Environ. Health A.

[109] Crespo-López M.E., Macêdo G.L., Pereira S.I.D., Arrifano G.P.F., Picanço-Diniz D.L.W., do Nascimento J.L.M., Herculano A.M. (2009). Mercury and human genotoxicity: Critical considerations and possible molecular mechanisms. Pharmacol. Res.

[110] Lee C.-H., Lin R.-H., Liu S.H., Lin-Shiau S.-Y. (1997). Distinct genotoxicity of phenylmercury acetate in human lymphocytes as compared with other mercury compounds. Mutat. Res.

[111] Pinheiro M.C.N., Macchi B.M., Vieira J.L.F., Oikawa T., Amoras W.W., Guimarães G.A., Costa C.A., Crespo-López M.E., Herculano A.M., Silveira L.C.L. (2008). Mercury exposure and antioxidant defenses in women: A comparative study in the Amazon. Environ. Res.

[112] Gupta R.S., Gupta E.S., Dhakal B.K., Thakur A.R., Ahnn J. (2004). Vitamin C and vitamin E protect the rat testes from cadmium-induced reactive oxygen species. Mol. Cells.

[113] Karbownik M., Gitto E., Lewinski A., Reiter R.J. (2001). Induction of lipid peroxidation in hamster organs by the carcinogen cadmium: Melioration by melatonin. Cell Biol. Toxicol.

[114] Cha S.H., Suh C.K. (2010). Heme oxygenase-1 mediated protective effect of methyl gallate on cadmium-induced cytotoxicity in cultured mouse mesangial cells. Mol. Cell. Toxicol.

[115] Park M.S., Shin H.S., Lee J.H., Kil G.-S., Choi C.Y. (2010). Influence of quercetin on the physiological response to cadmium stress in olive flounder, *Paralichthysolivaceus*: Effects on hematological and biochemical parameters. Mol. Cell. Toxicol.

[116] Fenoglio I., Corazzari I., Francia C., Bodoardo S., Fubini B. (2008). The oxidation of glutathione by cobalt/tungsten carbide contributes to hard metal-induced oxidative stress. Free Radic. Res.

[117] Salnikow K., Donald S.P., Bruick R.K., Zhitkovich A., Phang J.M., Kasprzak K.S. (2004). Depletion of intracellular ascorbate by the carcinogenic metals nickel and cobalt results in the induction of hypoxic stress. J. Biol. Chem.

[118] Stefaniak A.B., Harvey C.J., Bukowski V.C., Leonard S.S. (2010). Comparison of free radical generation by pre- and post-sintered cemented carbide particles. J. Occup. Environ. Hyg.

[119] O’Brien T., Mandel H.G., Pritchard D.E., Patierno S.R. (2002). Critical role of chromium (Cr)-DNA interactions in the formation of Cr-induced polymerase arresting lesions. Biochemistry.

[120] Quievryn G., Messer J., Zhitkovich A. (2002). Carcinogenic chromium(VI) induces cross-linking of vitamin C to DNA *in vitro* and in human lung 549 cells. Biochemistry.

[121] Quievryn G., Peterson E., Messer J., Zhitkovich A. (2003). Genotoxicity and mutagenicity of chromium(VI)/ascorbate-generated DNA adducts in human and bacterial cells. Biochemistry.

[122] de Flora S., Wetterhahn K.E. (1989). Mechanisms of chromium metabolism and genotoxicity. Life Chem. Rep.

[123] Hunaiti A.A., Soud M. (2000). Effect of lead concentration on the level of glutathione, glutathione *S*-transferase, reductase and peroxidase in human blood. Sci. Total Environ.

[124] Valko M., Leibfritz D., Moncol J., Cronin M.T.D., Mazur M., Telser J. (2007). Free radicals and antioxidants in normal physiological functions and human disease. Int. J. Biochem. Cell Biol.

[125] Batra N., Nehru B., Bansal M.P. (1998). The effect of zinc supplementation on the effects of lead in the rat testis. Reprod. Toxicol.

[126] Othman A.I., El Missiry M.A. (1998). Role of selenium against lead toxicity in male rats. J. Biochem. Mol. Toxicol.

[127] Pande M., Flora S.J.S. (2002). Lead induced oxidative damage and its response to combined administration of alpha-lipoic acid and succimers in rats. Toxicology.

[128] Schurz F., Sabater-Vilar M., Fink-Gremmels J. (2000). Mutagenicity of mercury chloride and mechanisms of cellular defence: The role of metal-binding proteins. Mutagenesis.

[129] Herculano A.M., Crespo-Lopez M.E., Lima S.M.A., Pincanço-Diniz D.L.W., do Nascimento J.L.M. (2006). Methylmercury intoxication activates nitric oxide synthase in chick retinal cell culture. Braz. J. Med. Biol. Res.

[130] Rao M.V., Chinoy N.J., Suthar M.B., Rajvanshi M.I. (2001). Role of ascorbic acid on mercuric chloride-induced genotoxicity in human blood cultures. Toxicol. In Vitro.

[131] Passos C.J.S., Mergler D., Fillion M., Lemire M., Mertens F., Guimarães J.R.D., Philibert A. (2007). Epidemiologic confirmation that fruit consumption influences mercury exposure in riparian communities in the Brazilian Amazon. Environ. Res.

[132] Myung S.-K., Kim Y., Ju W., Choi H.J., Bae W.K. (2010). Effects of antioxidant supplements on cancer prevention: Meta-analysis of randomized controlled trials. Ann. Oncol.

[133] Mahaffey K.R. (2005). Mercury exposure: Medical and public health issues. Trans. Am. Clin. Climatol. Assoc.

[134] Rojas M., Seijas D., Agreda O., Rodríquez M. (2006). Biological monitoring of mercury exposure in individuals referred to a toxicological center in Venezuela. Sci. Total Environ.

[135] Ronchetti R., Zuurbier M., Jesenak M., Koppe J.G., Ahmed U.F., Ceccatelli S., Villa M.P. (2006). Children’s health and mercury exposure. Acta Paediatr. Suppl.

[136] Tchounwou P.B., Ayensu W.K., Ninashvili N., Sutton D. (2003). Environmental exposure to mercury and its toxicopathologic implications for public health. Environ. Toxicol.

[137] Ho E. (2004). Zinc deficiency, DNA damage and cancer risk. J. Nutr. Biochem.

